# Oral flucloxacillin and phenoxymethylpenicillin versus flucloxacillin alone for the emergency department outpatient treatment of cellulitis: study protocol for a randomised controlled trial

**DOI:** 10.1186/1745-6215-14-164

**Published:** 2013-06-03

**Authors:** Michael Quirke, Abel Wakai, Peadar Gilligan, Ronan O’Sullivan

**Affiliations:** 1 Division of Population Health Sciences (PHS), Emergency Care Research Unit (ECRU), Royal College of Surgeons in Ireland, 123 St. Stephens Green, Dublin 2, Ireland; 2 Emergency Department, Beaumont Hospital, PO Box 1297, Beaumont Road, Dublin 9, Ireland; 3 Paediatric Emergency Research Unit (PERU), National Children’s Research Centre, Dublin 12, Ireland; 4 Department of Emergency Medicine, Cork University Hospital, Wilton, Cork, Ireland

**Keywords:** Cellulitis, Adult, Flucloxacillin, Phenoxymethylpenicillin, Penicillin V, Oral, Randomised controlled trial, Double blind, Emergency department

## Abstract

**Background:**

Oral flucloxacillin, either alone or in combination with phenoxymethylpenicillin, is a commonly prescribed antibiotic for the treatment of cellulitis, particularly in Ireland and the United Kingdom. This study aims to establish the non-inferiority of oral monotherapy (flucloxacillin alone) to dual therapy (flucloxacillin and phenoxymethylpenicillin) for the outpatient treatment of cellulitis in adults.

**Methods/design:**

This study is a multicentre, randomised, double-blind, placebo-controlled trial of adults who present to the emergency department (ED) with cellulitis that is deemed treatable on an outpatient basis with oral antibiotics. After fulfilling specified inclusion and exclusion criteria, informed consent will be taken. Patients will be given a treatment pack containing 7 days of treatment with flucloxacillin 500 mg four times daily and placebo or flucloxacillin 500 mg four times daily and phenoxymethylpenicillin 500 mg four times daily. The primary outcome measure under study is the proportion of patients in each group in which there is greater than or equal to a 50% reduction in the area of diameter of infection from the area measured at enrolment at the end-of-treatment visit (7 to 10 days). Secondary endpoints include a health-related quality of life measurement as rated by the SF-36 score and the Extremity Soft Tissue Infection Score (not validated), compliance and adverse events. Patients will be followed up by telephone call at 3 days, end-of-treatment visit (EOT) at 7 to 10 days and test-of-cure (TOC) visit at 30 days. To achieve 90% power, a sample size of 172 patients per treatment arm is needed. This assumes a treatment success rate of 85% with oral flucloxacillin and phenoxymethylpenicillin, an equivalence threshold Δ = 12.5% and an α = 0.025. Non-inferiority will be assessed using a one-sided confidence interval on the difference of proportions between the two groups. Standard analysis including per-protocol and intention-to-treat will be performed.

**Discussion:**

This trial aims to establish the non-inferiority of flucloxacillin monotherapy to dual therapy in the treatment of uncomplicated cellulitis among ED patients. In doing so, this trial will bridge a knowledge gap in this understudied and common condition and will be relevant to clinicians across several different disciplines.

**Trial registration:**

EudraCT Number 2008-006151-42

## Background

Cellulitis is an acute, subacute or chronic inflammation of the dermis and subdermal tissues usually caused by a bacterial infection [1]. Current use tends to regard the term erysipelas as a form of cellulitis rather than as a distinct entity in which a raised leading edge represents dermal inflammation alone [2]. Indeed, the terminology used to describe various types of skin and soft tissue infection (SSTI) is complicated because of the use of terms to describe different types of infection (cellulitis, erysipelas, abscess), clinical scenarios (Fournier gangrene) and aetiological agents (‘clostridial myonecrosis’) [3].

Cellulitis is an understudied condition and the numbers of patients seen and treated in Irish emergency departments (EDs) is unknown. In 2009 in Ireland it accounted for 3,877 hospital admissions resulting in a median hospital length-of-stay (LOS) of 4 days [4]. In the United Kingdom, one survey showed that it accounted for 3% of emergency visits to a district hospital [5] and results in approximately 69,576 hospital admissions annually [6]. In the Netherlands, cellulitis and erysipelas accounted for 3,500 hospitalisations in 1999 to 2001, with an average LOS of 12.1 days [7]. The population prevalence in the same year was 15.2/100,000 inhabitants, with ten times as many patients treated in the community as in hospital. In the era of community acquired methicillin resistant *Staphylococcus aureus* (CA-MRSA) infection in the United States, outpatient visits for purulent skin and soft tissue infection increased from 32.1 to 48.1 per 1,000 population between 1997 and 2005 [8] with a four-fold increase in hospitalisation rate for cellulitis/abscess between 1999 and 2005 [9]. Cellulitis and erysipelas cost on average €5,300 per hospitalised patient in the Netherlands in 1999 to 2001, with total costs of €13.7 million [7].

*Staphylococcus aureus* and β-haemolytic Streptococci Lancefield Group A, C and G are the most common causative agents of cellulitis [10]. In a recent systematic review examining the bacteriological etiology of cellulitis in intact skin, Chira *et al*. [11] examined 16 trials involving 808 patients. Only 16% had positive cultures, of which *Staphylococcus aureus* was almost twice as commonly isolated as Streptococcus Group A. This finding is especially relevant in North America where the onset of true CA-MRSA infection has reached epidemic proportions [8,10]. Enterococci are occasionally isolated, usually in patients with leg ulcers, and anaerobic bacteria are the least commonly isolated subgroup and include *Peptostreptococcus* species, *Bacteroides fragilis*, *Prevotella* species, *Porphyromonas* species and *Clostridium* species [1]. In clinical practice, the majority of these infections can be treated on an outpatient basis with European [12-14] and North American [15] prescribing guidelines recommending beta-lactam antibiotics active against gram-positive bacteria. Despite the increase in CA-MRSA, the Infectious Diseases Society of America (IDSA) also still recommends beta-lactam antibiotics for ‘non-purulent’ cellulitis [15].

Cellulitis is clinically apparent as an area of spreading erythema characterised by warmth, pain and tenderness to palpation. It is on a spectrum of disease from mild infections, easily treatable with oral antibiotics, to severe necrotizing infections with associated high mortality. However, most people are not seriously ill and have a low risk of complications [16].

### Measures of severity

There are no internationally accepted criteria for ‘mild, moderate and severe’ cellulitis although this classification scheme is widely used in clinical practice [1]. The classification used by Eron *et al*. [16], (Table 1) is useful for guiding treatment decisions for patients with cellulitis. It has been used in local National Health Service (NHS) policy guidelines [17] and is recommended by the Clinical Resource Efficiency Support Team (CREST) group in Northern Ireland in its consensus statement on the management of cellulitis [5].

**Table 1:** **Eron classification system for patients with skin and soft tissue infection**[16]

**Class**	**Patient criteria**
**1**	Afebrile and healthy, other than cellulitis
**2**	Febrile and ill appearing, but no unstable comorbidities, or systemically well with comorbidity (PVD, CVI, morbid obesity)
**3**	Toxic appearance, or at least one unstable comorbidity, or a limb-threatening infection
**4**	Sepsis syndrome or life-threatening infection, for example, necrotizing fasciitis

*CVI* chronic venous insufficiency, *PVD* peripheral vascular disease.

The single ‘best antibiotic’ for treating cellulitis is unknown [1] and thus there is considerable variability in the class, dose and regimen of antibiotic prescribed [18]. Flucloxacillin either alone or in combination with phenoxymethylpenicillin or its intravenous form, benzylpenicillin, are commonly prescribed in Great Britain and Ireland [19,20]. Various sources including authoritative textbooks [21], the national prescribing formulary in the United Kingdom [22] and local guidelines, provide conflicting information on whether monotherapy with flucloxacillin or dual therapy with combined flucloxacillin and phenoxymethylpenicillin is optimal, particularly for cellulitis treatable on an outpatient basis. Recent evidence has shown that oral phenoxymethylpenicillin prevents cellulitis recurrence in patients with two or more episodes of leg cellulitis [23].

A study comparing the efficacy, tolerability and compliance of combined oral flucloxacillin and phenoxymethylpenicillin with flucloxacillin alone in the outpatient treatment of cellulitis would provide a valuable resource for clinicians, and may close a gap in the research-based knowledge of this condition.

## Methods/design

### Study aims

To determine whether flucloxacillin monotherapy is non-inferior to combined flucloxacillin and phenoxymethylpenicillin (dual therapy) in the management of Eron Class I and II cellulitis.

### Study design and setting

It is intended to carry out a phase 4, multicentre, active-controlled, double-blind, non-inferiority randomised trial in patients attending the ED with cellulitis deemed clinically treatable with an oral antibiotic as an outpatient (Eron Class I and II cellulitis). Patients will be randomized to receive either flucloxacillin and phenoxymethylpenicillin or flucloxacillin and placebo in a 1:1 ratio.

### Ethical considerations

This study is to be performed in accordance with the Good Clinical Practice (GCP) Guidelines, the EU CT Directive 2001/20.EC, GCP Commission Directive 2005/28/EC, the Declaration of Helsinki (2008) and with all other local regulatory requirements. Risk Analysis was carried out as part of protocol development. The study protocol was approved by Beaumont Hospital Research and Ethics Committee. Patients will be enrolled from the ED they attend. This may be by self-referral or general practitioner (GP) referral.

### Regulatory considerations

The Irish Medicines Board (IMB) is the competent authority for the review and approval of clinical trials with an investigational medicinal product (IMP) in Ireland. The application process for a medicinal trial was successful after formal engagement with the IMB prior to application and during the application process. IMB approval for this trial was granted on 30 January 2009.

### Subject selection

Patients more than 16 years of age satisfying the inclusion and exclusion criteria (Table 2) who present to the recruiting EDs with cellulitis during the study period, will be eligible for recruitment into the trial. All potential patients will be given time to read the patient information leaflet prior to informed consent being taken. Once consent is signed, the enrolment procedure will continue to random selection of the drug pack. Enrolment will be undertaken by any of the following: a hospital doctor with minimum 2 years experience in Emergency Medicine (including consultants, specialist registrars, associate specialists and non-specialist registrars) and Advanced Nurse Practitioners who are registered prescribers of medications.

**Table 2:** Inclusion and exclusion criteria

**Inclusion criteria**	**Exclusion criteria**
>16 years of age	Any cellulitis that requires intravenous antibiotic
Cellulitis of any body area excluding the perineum, that is treatable with oral antibiotic as an outpatient and in whom either combination of antibiotic is likely to produce a clinical response	Any cellulitis worse than Eron Stage 2 cellulitis
Any two of the following signs:	Pregnancy
1. Erythema	Lactation
2. Warmth	Hypersensitivity to penicillin
3. Tenderness	Chronic skin condition at the site of infection
4. Swelling	Infection involving prosthetic material
5. Regional lymphadenopathy	Thermal injury
Fluency in written and spoken English	Acne vulgaris
	Perineal cellulitis (high risk of anaerobic infection)
	Fungal infection of scalp or nail bed
	Immunodeficiency
	Severe renal or hepatic dysfunction
	Concomitant treatment with oral, parenteral or topical antibiotics at infection site
	Patients taking systemic corticosteroids at a dose exceeding 15 mg (or equivalent) per day for more than 7 days
	Refusal or inability to give informed consent

### Inclusion and exclusion criteria

Inclusion and exclusion criteria are presented in Table 2.

### Patient randomisation

The randomisation process has been designed by CSTAR (Health Research Board Centre for Support and Training in Analysis and Research), Dublin, Ireland. Trial randomisation codes will be generated by CSTAR. It is intended to use block randomization using a permuted block design with a computer random number generator without stratification, to create two treatment groups of 172 patients (n = 344).

### Randomisation treatments

#### Allocation concealment

A sequentially numbered opaque sealed envelope (SNOSE) will be opened for each patient who consents to enrollment into the study. This envelope will be opaque to intense light. It will contain a copy of the information leaflet, a consent form, an SF-36 and ESTI score, a paper tape measure, the study drugs in two amber containers, and a patient proforma with a unique pre-printed patient identifier to be used throughout the study. The proforma will be inserted in to the ED notes and scanned or stored securely as is standard practice with patient data in the EDs in question. The proforma will then be re-used for the end-of-treatment (EOT) and test-of-cure (TOC) visits. The SNOSEs will be stored in the secure Specialist Registrar Office available in each location, which will be accessible at all times. All study drugs will be packaged by the clinical trial pharmacist who will remain independent of all further patient treatments.

#### Drug appearance

Phenoxymethylpenicillin appears as a circular round white tablet with a marking ‘PEN 500’ on the side (SmPC Athlone Laboratories Limited, Ballymurray, Co. Roscommon, Ireland). The placebo formulation to be used will also be a white round tablet with a notch and contain lactose monohydrate, magnesium stearate and cellulose (FAGRON GmbH & Co. KG Von-Bronsart-Straße 12, 22885 Barsbüttel, Germany. http://www.fagron.de).

Flucloxacillin appears as a caramel coloured, hard capsule marked with ‘FXN 250’ or ‘FXN 500’. Since both groups are receiving flucloxacillin, product blinding is irrelevant.

All efforts will be made to ensure placebo and phenoxymethylpenicillin tablets are similar in appearance; it should be possible to have a dummy inscription on the placebo tablet.

#### Product liability

Public and product liability is in place for licensed medications issued by Athlone Laboratories and includes cover for the generic brands of phenoxymethylpencillin and flucloxacillin to be used in the study.

### Breaking of the study blind

#### On study

If an adverse event is regarded as a potential Serious Unexpected Suspected Adverse Reaction (SUSAR) by the sponsor the treatment group to which the trial subject affected belongs will be unblinded for that subject alone. The procedure will ensure that the identity of the investigational medicinal product (IMP) is only revealed as far as necessary (GCP Directive). All staff will have received training on all aspects of the trial protocol prior to commencement of the trial.

The principal investigator (PI) or authorised member of the team will have a written procedure for requesting randomisation codes for rapidly identifying a blinded IMP in an emergency. Breaking the blind of a trial subject will be at the discretion of the PI, when clinically indicated for the safety of the patient, or in the event of a SUSAR. If the patient needs to be unblinded we will refer to the unblinding SOP for complete details of the procedures to be followed. The master randomisation codes will be kept by the clinical trial Pharmacist and the PI. Unblinding will be performed by the senior clinician/pharmacist when the criteria for a Serious Adverse Event (SAE/SUSAR) have been met and there is a necessity for the PI or treating healthcare professional to know which treatment the patient is receiving to ensure that the patient receives appropriate urgent safety measures.

A 24-hour contact number will be available in the circumstances when unblinding is required. The scenario will be communicated and when the unblinding criteria are met the unblinding will ensue. The PI will document the breaking of the code, and the reasons for doing so, in the site file and in the patient’s medical notes, and in accordance with the clinical trial protocol. If at the EOT visit a clinical failure is apparent and the addition of phenoxymethylpenicillin is felt to be clinically necessary, code breaking will be made possible on a 24-hour basis by making contact with the PI. It is felt that unblinding will be an extremely unlikely event as a treatment failure will more than likely require intravenous antibiotics. Any compromises in blinding will be reported in the trial conclusion.

#### Following completion of the study

Study unblinding will only take place once the statistical analysis plan has been agreed by the trial team and the final database has been locked.

### Study procedure

#### Interventions

##### Wound/skin care

Standard wound care for cellulitis will be permissible in both groups and may include saline soaks and non-adherent dressings. The area of infection will be drawn with a waterproof skin marker and the maximal diameter of the area of infection measured and recorded.

##### Microbiological assay

Whenever possible a specimen for culture and sensitivity will be taken from the infection site at enrolment. This may be from blister fluid or exudate. Specimen results will be made available at follow-up to guide appropriate therapy. Blood cultures will not be taken in this study. Since the expected microbiological yield will be low, a test of microbiological cure will not be used in this study.

##### Therapeutic intervention

Once enrolled patients will be given a 7-day course of either oral flucloxacillin 500 mg four times daily and oral phenoxymethylpenicillin 500 mg four times daily or oral flucloxacillin 500 mg four times daily and oral placebo four times daily.

##### Follow-up

Patients will be telephoned at 3 to 5 days by a study investigator to encourage treatment compliance and EOT visit follow-up. Any deterioration in patient symptoms may be addressed at this visit and a return for clinical assessment may be recommended. The patient will be reviewed at 7 to 10 days after treatment commencement (EOT visit) in the enrolment centre and the area of infection will be measured. At this visit, the patient will be given an SF-36 and ESTI score to complete. A final TOC visit or telephone call will be arranged for 30 days after the enrolment of the patient into the trial.

#### Outcome measures

##### Primary outcome

The primary outcome measure under study is the proportion of patients treated in each group in which there is greater than or equal to a 50% reduction in the maximal diameter of erythema from the area measured at enrolment to that at the end-of-treatment visit (7 to 10 days).

##### Measurement of the primary outcome

Clinical cure: Reduction in the area of cellulitis by ≥50% of the initial diameter.

##### Secondary outcomes

•Clinical failure: a failure of the initial maximal diameter of infection to reduce by ≥50%

•Clinical relapse: initial improvement in cellulitis at the EOT visit followed by a worsening or re-appearance of cellulitis at the TOC visit.

•Quality of life: the SF-36 and the ESTI score at the EOT visit will be measured.

•Compliance with therapy: the number of unused study medications counted at the EOT visit will be measured.

•Adverse Events: all adverse events will be recorded and submitted to the Irish Medicines Board and the Research and Ethics Committee.

•Microbiological profile: the results of specimens sent for culture to receiving laboratories will be profiled.

### Sample size, power and statistical methods

Non-inferiority of oral flucloxacillin and placebo relative to oral flucloxacillin and phenoxymethylpenicillin for the above primary and secondary outcomes will be assessed. Sample size per treatment arm is calculated according to an assumed treatment success rate of 85% with oral flucloxacillin and phenoxymethylpenicillin, an equivalence threshold Δ = 12.5% and an α = 0.025 (as this is a non-inferiority study). Sample sizes were calculated using PASS statistical software (Hintze J.(2008) PASS 2008. NCSS, LLC, Kaysville, Utah, USA http://www.ncss.com). Given the preferred study power of 90% for non-inferiority trials, a minimum sample size of 172 in each treatment group is required (n = 344). Non-inferiority will be assessed using a one-sided confidence interval (CI) on the difference of proportions between the two groups. If the upper limit of the CI is less than the equivalence threshold of 12.5%, then non-inferiority is inferred.

#### Data analysis

Both intention-to-treat (ITT) and per-protocol analysis will be performed. ITT analyses include all patients randomised to the trial regardless of whether they satisfied entry requirements, received the assigned treatments, withdrew from the trial or adhered to the protocol. Missing values will be imputed using a suitable imputation method (last value carried forward or mixed model). A per-protocol analysis includes only patients who completed the full course of treatment and adhered to the protocol requirements. In a non-inferiority trial setting a per-protocol analysis may be more appropriate since it is more likely to reflect actual differences between the two treatments. Furthermore, ITT analysis may be interpreted as being too liberal in a non-inferiority trial and may bias toward making the two treatments appear similar [24]. As a result, both an ITT and per-protocol analysis will be performed on the resulting data to assess non-inferiority of the placebo/flucloxacillin combination. In particular, to declare non-inferiority, both ITT and per protocol analysis should exclude the non-inferiority margin.

#### Bias and confounding variables

In terms of selection bias, we feel that this study targets a patient population to whom this research ultimately will be clinically applicable and valuable. Every effort will be made to ensure that recruitment of participants occurs over all 24-hour periods (including weekends) by having patients recruited by the ED physician treating the patient. We anticipate that the randomised, double-blinded, controlled design of this study will minimise the effect of confounding variables on our analysis.

#### Safety reporting

All adverse events that occur during the study period observed by one of the clinical staff, or reported by the patient or parent/guardian spontaneously, or in response to a direct question, will be noted on the appropriate form (that is, Adverse Event (AE), SAE or SUSAR form). These forms are de-identified. The following procedures will take place depending on the type of event that has occurred.

##### Adverse event

Each AE will be recorded by a member of the research team on an AE Form. Adverse events will be classified on the form in terms of their severity, association with the study drug, expectedness and seriousness. They will be recorded on an Adverse Event Log. The adverse events will be reported to the sponsor and to the institutional Health Research Ethics Committee (HREC) on a yearly basis as part of an annual safety report, and at the end of the trial.

##### Serious adverse event

Each SAE will be recorded by a member of the research team on an SAE Form. SAEs will be classified on the form in terms of their severity, relatedness to the study drug, and expectedness. They will be recorded on a Serious Adverse Event Log. All SAEs will be reported on the SAE form within 24 hours to the sponsor and the institutional HREC. The research team will ensure that follow-up information and a detailed written report are provided when available.

##### Suspected unexpected serious adverse drug reactions

Each SUSAR will also require expedited reporting to the sponsor. This will occur as soon as possible, but no later than 24 hours after a member of the research team has first knowledge of the minimum criteria for expedited reporting. In each case, relevant follow-up information will be sought and a detailed, written report completed as soon as possible. The sponsor has responsibility to ensure all relevant and available information is forwarded to the competent authority (Irish Medicines Board) and the appropriate HREC. For fatal or life-threatening events this will be done as soon as possible and not later than seven days after the sponsor becomes aware of the event. Additional relevant information will be sent within eight days of the first report. This will be sent no later than an additional 15 calendar days. For AEs that are not fatal or life threatening, the sponsor will ensure that a SUSAR is reported as soon as possible and in any event not later than 15 days after the sponsor is first aware of the event.

The study participants will be provided with 24-hour contact details of a study representative if they have concerns about any component of the study.

## Trial status

This trial requires further funding in order to commence enrolment.

## Abbreviations

AE: Adverse event; CA-MRSA: Community acquired methicillin resistant *Staphylococcus aureus*; CI: Confidence interval; CSTAR: Centre for support and training in analysis and research; CREST: Clinical resource efficiency support team; ED: Emergency department; EOT: End-of-treatment; GCP: Good clinical practice; GP: General practitioner; HREC: Health research ethics committee; IMB: Irish medicines board; ITT: Intention to treat; LOS: Length of stay; NHS: National health service; PI: Principal investigator; SAE: Serious adverse event; SNOSE: Sequentially numbered opaque sealed envelope; SOP: Standard operating procedure; SSTI: Skin and soft tissue infection; SUSAR: Suspected unexpected serious adverse reaction; TOC: Test-of-cure.

## Competing interests

The authors declare that they have no competing interests.

## Authors’ contributions

MQ, PG, AW and ROS conceived the study and each made substantial contributions to design; all have been involved in drafting the manuscript and revising it critically for intellectual content; and have given final approval of the version to be published.
